# Effectiveness of an Educational Intervention on Status Epilepticus Among the Caregivers of Children With Epilepsy: An Interventional Study

**DOI:** 10.7759/cureus.40735

**Published:** 2023-06-21

**Authors:** Riyadh W Alharbi, Ahmed Kaki, Sadia Tabassum

**Affiliations:** 1 Pediatric Neurology, Prince Sultan Military Medical City, Riyadh, SAU; 2 Pediatric Neurology, King Fahad Medical City, Riyadh, SAU

**Keywords:** status epilepticus, saudi arabia, intervention, knowledge, emergency, caregivers

## Abstract

Background

Status epilepticus (SE) is one of the most common and well-known neurological emergencies in pediatrics, especially among kids under two years of age. Early identification and treatment are crucial in the prevention of neurological complications and morbidities. This study aimed to assess the effectiveness of an educational intervention about SE among caregivers of pediatric patients with epilepsy.

Methodology

This interventional study was conducted among a convenient sample of 206 caregivers of pediatric patients with epilepsy in King Fahad Medical City, Riyadh, Saudi Arabia, from November 2020 to July 2021. We included patients who were aged 14 years or less and received rescue medication prescriptions in 2020. The educational intervention was applied to caregivers, and knowledge was compared before and after the intervention. A self-administered questionnaire was utilized.

Results

The mean (±SD) age of children was 7.5 (±3.7) years. The mean (±SD) age of caregivers was 36.4 (±6.1) years. About 51.5% of the children were males. The majority of caregivers were mothers (89.8%). The mean (±SD) score of total knowledge was 12.3/20 (±2.6) before the intervention which increased to 15.7/20 (±3.1) after the intervention, and the difference was found to be statically significant (p = 0.001). This indicates that the educational intervention was effective.

Conclusions

The educational intervention administered in this study significantly improved the knowledge of caregivers of pediatric patients with epilepsy and can help in providing better care to the patients.

## Introduction

Epilepsy is a common neurological disorder in Saudi Arabia [1]. The prevalence of epilepsy in Saudi Arabia is estimated to be 6.45 per 1,000 [1]. Status epilepticus (SE) is one of the well-known neurological emergencies in pediatrics, especially in children under two years of age. Early identification and treatment are crucial in the prevention of neurological complications [2-5] and long-term morbidities. SE is defined as any seizure that lasts for more than five minutes in duration or the occurrence of two or more seizures without gaining the baseline conscious level [2]. The annual incidence of SE worldwide is 10-58 per 100,000 population, and the mortality is about 2.7-22% globally [3]. One of the most critical factors in prognosis is the early identification and treatment of SE at home or in public places, as early intervention is a good prognostic factor [2-6]. There are multiple choices for prehospital abortive medications such as rectal diazepam and buccal midazolam which are widely used in Saudi Arabia [2,5]. Although both medications have similar efficacy, buccal midazolam is socially more accepted [7].

The awareness of the caregivers and emergency medical staff about the identification of SE and the use of rescue medications can lead to better outcomes and will decrease the harmful effect of SE in patients. To our knowledge, there is no previous interventional study that measured the knowledge in SE, however; an Iranian study focused on educational interventions for febrile seizures.

This study hypothesizes that an educational intervention will improve the knowledge of the participants (caregivers) in the identification and management of SE. This study aims to assess the effectiveness of an educational intervention about SE among the caregivers of pediatric epileptic patients.

## Materials and methods

Study design and settings

This interventional study was conducted among a convenient sample of 206 caregivers of pediatric epileptic patients in King Fahad Medical City (KFMC), Riyadh, Saudi Arabia, from November 2020 to July 2021. We included patients who were aged 14 years or less, were followed in the Paediatric Neurology Clinic and Emergency Room, and received rescue medication in 2020. Those who were aged more than 14 and did not receive rescue medications were excluded. The sample size was calculated to be 190 by using the Epi Info program. To compensate for non-response, 10% was added to the sample. Hence, the total sample size was 219.

Study instruments

A self-administered questionnaire was used which included three parts. The first part included questions about the sociodemographic data of the child and the caregiver such as gender, age, educational level, and type of care provider. The second part included questions about the emergency medicine used for the child, the staff who explained the use of the emergency medicines, and the obstacles that prevented the caregiver from using emergency medicines. The third part included questions about the knowledge of the caregivers about epilepsy and SE. The questionnaire was designed and face validated by a team of two neurologists and two preventive medicine consultants, according to the literature and guidelines [4,6,7]. It included questions on the definition of SE, its symptoms and signs, the proper management, and the appropriate use of emergency medications. It consisted of 14 items in which some items were answered with yes or no and some others were answered by selecting the true/false choices. Each true answer was coded one and each wrong answer was coded zero. The score was summed to obtain the total score of knowledge for each participant (minimum = 0, maximum = 20). The questionnaire was sent to the participants electronically through WhatsApp after obtaining their phone numbers from the records.

Intervention

An educational brochure was designed and validated by the research team based on the clinical guidelines and literature. The brochure included information on epilepsy, SE, and management and was illustrated by many figures. The brochure included information such as the difference between seizures and epilepsy, types of seizure and their symptoms, which measures to be taken when the seizure begins (with illustration), positioning the patient during seizures, illustrated procedures for giving diazepam injection or midazolam, and the side effects of diazepam injection and midazolam.

Data collection procedure

Data were collected by a team of trained residents and interns who are working in the Paediatric Neurology Department in King Fahad Medical City, Riyadh. The training was conducted by the main researcher. After obtaining the agreement and consent of the caregivers to participate in the study, the following steps were employed: (1) First step (pre-intervention): at baseline, the questionnaire was given to the participants to measure baseline knowledge and awareness. Each participant was recognized by their telephone number. (2) Second step (intervention): each participant was sent the brochure to read and understand via WhatsApp. (3) Third step (post-intervention): after one month, participants were contacted by phone and sent the same questionnaire to assess improvement in knowledge and awareness. The questionnaire was sent by WhatsApp. For each participant, answers for pre and post-intervention were entered into the SPSS program.

Pilot study

A pilot study was conducted among 20 caregivers who were not included in the main study. Those caregivers were recruited from outpatients and were sent the materials through WhatsApp after their permission. They were contacted by the research team after three days to obtain their feedback. Piloting was conducted for both the questionnaire and the brochure to ensure the clarity and understandability of the tools. Every participant was asked to give their opinion about the unclear points and how to improve clarity. A few points were modified according to the participants’ feedback.

Statistical analysis

Statistical analysis was performed using SPSS version 20 (IBM Corp., Armonk, NY, USA). Categorical variables were described as frequency and percentage, while continuous variables were described as mean ± SD. For the knowledge items, the correct answer was given a score of 1, and the wrong answer was scored zero. The total score of knowledge was obtained by summation of the score for each item. The total score of knowledge was normally distributed. Paired t-test was used to test the difference in total knowledge scores before and after the intervention. A two-sample t-test was used to compare the knowledge score between the sociodemographic variables. The chi-square test was used to compare the percentage of correct answers for each item separately before and after the intervention. The accepted level of significance was set below 0.05 (p < 0.05).

## Results

Sociodemographic characteristics of the respondents

Out of 219 caregivers who were asked to participate, 206 agreed to participate in this study (response rate = 94%). The mean (±SD) age of children was 7.5 (±3.7) years and ranged from 1 to 15 years. The mean (±SD) age of caregivers was 36.4 (±6.1) years and ranged from 24 to 68 years. About 35.4% of children were aged ≤5 years, 39.3% were aged 6-10 years, and 25.2% were aged 11-15 years. About 51.5% of the children were females and 48.5% were males. The majority of healthcare providers were mothers (89.8%). Overall, 58.3% of the participants had a university level of education (Table 1).

**Table 1:** Sociodemographic characteristics of the respondents.

	N	%
Gender of the child		
Male	100	48.5
Female	106	51.5
Age of the child (years)		
≤5	73	35.4
6–10	81	39.3
11–15	52	25.2
Age of the caregivers (years)		
<40	149	72.3
≥40	57	27.7
Caregivers		
Mother	185	89.8
Father	14	6.8
Others	7	3.4
Education of the healthcare provider		
School	86	41.7
University	120	58.3

The most common emergency medicine used for the children was rectal diazepam (69.9%), followed by buccal midazolam (8.3%). The majority reported that physicians explained to them how to use emergency medicine (81.1%) during their clinic and emergency room visits. The most common obstacles that prevented the participants from using emergency medicines were “No availability” (30.1%), “I am afraid of its side effects” (22.3%), “Others” (18.4%), and “I do not know when to give it” (14.1%) (Table 2).

**Table 2:** Descriptive variables of the knowledge items.

	N	%
Which emergency medicine was used for your child?		
Rectal diazepam	144	69.9
Buccal midazolam	17	8.3
Nothing	45	21.8
Who explained how to use emergency medicines?		
Physician	167	81.1
Nurse	13	6.3
Pharmacist	17	8.3
Others	9	4.4
What are the obstacles that prevent you from using emergency medicines?		
I do not know when to give it	29	14.1
I do not know how to give it	14	6.8
Its harms outweigh its benefits	10	4.9
I am afraid of its side effects	46	22.3
No benefits	9	4.4
No availability	62	30.1
Others	38	18.4
Nothing	39	18.9

Level of knowledge before and after the intervention

The mean (±SD) score of total knowledge was 12.3 (±2.6) before the intervention. After the intervention, it increased to 15.7 (±3.1) (p = 0.001). This indicates that the intervention was effective (Table 3).

**Table 3:** Comparison of the knowledge score before and after the intervention.

	Mean knowledge	SD	P-value
Before intervention	12.3	2.6	0.001
After intervention	15.7	3.1	

Relationship between the knowledge score before the intervention and sociodemographic factors

There was no significant relationship between the knowledge score and sociodemographic factors before intervention (all p-values are >0.05). This indicates that the knowledge score before intervention is not affected by sociodemographic variables (Table 4). 

**Table 4:** Relationship between the knowledge score of caregivers before the intervention and sociodemographic factors.

	Mean	SD	P-value
Gender of the child			
Male	12.19	2.77	0.229
Female	12.66	2.93	
Age of the child (years)			
≤5	12.41	2.90	0.994
6–10	12.37	2.72	
11–15	12.42	3.14	
Age of caregivers (years)			
<40	12.69	2.59	0.078
≥40	11.63	3.44669	
Caregiver			
Mother	12.42	2.88	0.577
Father	11.71	3.07	
Others	13.00	2.70	
Education of caregivers			
School	12.18	2.99	0.099
University	12.55	2.81	

Relationship between the knowledge score after the intervention​​​​​​​ and sociodemographic factors

There was no significant relationship between the knowledge score and sociodemographic factors after intervention (all p-values are >0.05). This indicates that the knowledge score after the intervention is not affected by sociodemographic variables (Table 5).

**Table 5:** Relationship between the knowledge score of caregivers after the intervention and sociodemographic factors.

	Mean	SD	P-value
Child gender			
Male	16.25	3.00	0.505
Female	15.22	3.19	
Age of the child (years)			
≤5	15.31	3.17	0.511
6–10	16.38	2.78	
11–15	15.26	3.46	
Age of the caregivers (years)			
<40	15.54	3.08	0.179
≥40	16.19	3.25	
Caregivers			
Mother	15.84	2.97	0.266
Father	14.64	4.95	
Others	14.71	2.62	
Education of the caregivers			
School	16.18	2.66	0.071
University	15.39	3.40	

The difference in response to knowledge items before and after the intervention

Table 6 shows the percentages of correct answers for each item of knowledge before and after the intervention. In 15 items out of 20, there was a statistically significant increase in the correct answer after the intervention. For example, for the item “Status epileptics is defined as a seizure that lasts more than one minute,” the correct answer was reported by 46.1% before the intervention, which increased to 81.1% after the intervention (p < 0.001).

**Table 6:** Difference in response to knowledge items before and after the intervention.

Questions	Percentage of correct answers		P-value
	Pre-intervention	Post-intervention	
1. Status epileptics is defined as a seizure that lasts for more than one minute (correct answer: no)	46.1	81.1	<0.001
2. Is a prolonged seizure necessary to diagnose status epilepticus with recurrent episodes within a short time? (correct answer: no)	57.3	77.7	<0.001
3. Can status epilepticus occur without apparent physical symptoms such as convulsions (correct answer: yes)	63.6	80.6	<0.001
4. What to do when fits start? (correct answer: lay the child on one side).	87.9	95.6	0.003
5. What to do if the child has a prolonged epileptic seizure? (correct answer: give emergency TTT then go to hospital)	66.0	81.6	<0.001
6. What is the right time to give emergency medicines (correct answer: after five minutes)	63.1	81.6	<0.001
7. When to take the child to the hospital			
7.1. The child has not returned to the position before the seizure (correct answer: yes)	71.4	85.9	<0.001
7.2. Immediately after fits stop	89.8	92.7	0.295
7.3: Long time fit (correct answer: yes)	70.4	85.0	0.001
7.4. The child returned to normal	98.1	98.5	0.678
7.5. Allergic reaction (correct answer: yes)	35.4	69.9	<0.001
7.6. Failure of two doses to stop fits (correct answer: yes)	49.0	73.8	<0.001
7.7. Before giving emergency treatment	94.2	97.1	0.148
8. What do you think about emergency medicine (correct answer: safe)	52.4	78.2	<0.001
9. Where are emergency medicines kept? (correct answer: refrigerator)	25.7	58.7	<0.001
10. Do you know the expiration date of your emergency medications?	83.5	90.8	0.027
11. If the medicine is expired and your child has a seizure, can you give him the treatment? (correct answer: no)	90.8	94.2	0.191
12. Do you take emergency medicines with you when going to public places or when traveling (correct answer: yes)	86.9	90.3	0.278
13. Do you know how to use emergency medicines?	86.9	95.6	0.002
14 If you give your child a dose of emergency medicine and the seizure does not stop, do you give another dose? (correct answer: yes)	21.4	54.9	<0.001

## Discussion

This study aims to assess the effectiveness of an education intervention on SE and the use of rescue medications among caregivers of epileptic children in King Fahad Medical City. Literature has suggested that administering appropriate educational or behavioral interventions to caregivers would significantly improve their knowledge, willingness, and confidence to handle epileptic children during acute situations [8,9]. Consistent with this postulation, this study found caregivers’ knowledge of SE and the use of emergency rescue medications was significantly improved at post-educational intervention. This finding was consistent with a previous study from Iran that showed the effectiveness of an educational program to significantly improve knowledge of handling epileptic emergencies among caregivers of children with febrile seizures [10]. Similarly, another cohort that comprised preschool teachers from Germany reported better knowledge and increased willingness to manage acute seizures in children after being provided with an educational teaching session [11].

Methods of health information delivery through educational interventions usually ranged from conventional modes (leaflets, brochures, or instructor-delivered programs) to sophisticated online resources (podcasts, webinars, or health apps), with each delivering a different level of effectiveness influenced by the population’s demography, study design, or time gaps of interventions being administered from baseline to follow-ups [12]. This study found that improvement in knowledge among caregivers of epileptic children was independent of the sample’s demographic characteristics. This finding was consistent with a previous Malaysian study that showed improvement in knowledge of epilepsy among parents with epileptic children using an animated feature of an information technology (IT)-based epilepsy education program [13]. However, the current study findings were in contrast with previous works from the Middle East that found a caregiver’s knowledge of a child’s epilepsy to be associated with employment status or education levels [10,14]. Plausible explanations of insignificant relationships in this study could be attributed to the fact that all caregivers were literate, thus they would have been able to appreciate the information provided via self-apprehended brochures compared to those with varying literacy levels who may require assisted teaching approach for knowledge improvement.

SE, a prolonged seizure lasting for more than five minutes requires urgent attention and intervention by caregivers [15]. The administration of common emergency rescue medications in a timely and correct manner is essential to prevent serious complications such as hypoxic insult, aspiration, recurrent SE, or mortality in an epileptic child [16]. But before the administration of these rescue medications, caregivers’ of the child would need to have good knowledge about the disease, symptoms, and the actions required to identify and abort such neurological emergencies in a pre-hospital setting. To date, although pre- and post-study designs are limited to evaluate consistencies in the literature, most baseline studies or cross-sectional surveys have reported low-to-moderate levels of caregivers’ knowledge about the disease and interventions required to abort seizures [17-21]. These studies fundamentally pointed out the need for educational and behavioral interventions for caregivers of an epileptic child to execute preventive and control measures during acute situations.

This study found that caregivers’ knowledge of identifying SE (epilepsy of a prolonged duration), the symptoms of the disease, and what actions needed to be taken (administration of emergency rescue medications, observation to recovery position, or the need to urgently transport the child to the hospital if treatment fails) were significantly improved at post-intervention. This finding was consistent with previous studies conducted among parents of epileptic children in Austria and Germany [22], families of epileptic children in the United States [16], and preschool teachers in Germany [8].

A child with a history of epilepsy or SE will usually be prescribed rescue medications. However, timely administration of rescue medications by caregivers is crucial to prevent complications. The administration of rescue medications among caregivers is principally dependent on the training provided by the healthcare provider, knowledge about the onset of fits or duration of SE, appropriate dosage of medications, and observation of the child [16]. This study found that these aspects of knowledge were significantly improved post-intervention among the caregivers. Most caregivers were trained by physicians on how to use emergency rescue medicines, consistent with the study reported by Gainza-Lein et al. (2017) [16]. However, the main barrier that caused caregivers not to administer rescue medications in this study was “availability.” This reason was in contrast with a previous study that only reported a minority of parents not administrating the drugs [16]. “Fear of side effects” was the second highest reported barrier for caregivers to administer rescue medications in this study, consistent with a recent study from Turkey [23].

Study limitations

The limitations of this study need to be acknowledged. Given the nature of the pre- and post-test intervention design, this study was limited by the absence of a control group; hence, it was not sufficiently powered to determine the effect size of the associations between predictors and knowledge improvement. The timing of the assessment of post-test intervention is crucial, as it can lead to to recall bias among the participants.

## Conclusions

The educational intervention administered in this study significantly improved the knowledge of caregivers of epileptic children. Although most caregivers were prescribed emergency rescue medications for their children, there were some barriers to administration such as “fear of side effects.” Future studies or interventions can incorporate behavioral and psychological modules within educational programs to boost confidence among caregivers to administer rescue medications timely without fear to abort a child’s SE.
